# Influence of Serotonin Transporter Gene Polymorphism (5-HTTLPR Polymorphism) on the Relation between Brain 5-HT Transporter Binding and Heart Rate Corrected Cardiac Repolarization Interval

**DOI:** 10.1371/journal.pone.0050303

**Published:** 2013-01-14

**Authors:** Esa Kauppila, Esko Vanninen, Salla Kaurijoki, Leila Karhunen, Kirsi H. Pietiläinen, Aila Rissanen, Jari Tiihonen, Ullamari Pesonen, Jaakko Kaprio

**Affiliations:** 1 Department of Clinical Physiology and Nuclear Medicine, Kuopio University Hospital and University of Eastern Finland, Kuopio, Finland; 2 Department Clinical Physiology, North-Karelia Central Hospital, Joensuu, Finland; 3 Department of Clinical Nutrition, Institute of Public Health and Clinical Nutrition, University of Eastern Finland, Kuopio, Finland; 4 Obesity Research Unit, Department of Medicine, Division of Endocrinology, Helsinki University Central Hospital and University of Helsinki, Helsinki, Finland; 5 Institute for Molecular Medicine Finland, FIMM, University of Helsinki, Helsinki, Finland; 6 Obesity Research Unit, Department of Psychiatry, Helsinki University Hospital, Helsinki, Finland; 7 Department of Public Health, Hjelt Institute, University of Helsinki, Helsinki, Finland; 8 Department of Forensic Psychiatry, University of Eastern Finland, Niuvanniemi Hospital, Kuopio, Finland; 9 Department of Clinical Neuroscience, Karolinska Institutet, Stockholm, Sweden; 10 Department of Mental Health and Substance Abuse Services, National Institute for Health and Welfare, Helsinki, Finland; 11 Department of Pharmacology, Drug Development and Therapeutics, University of Turku, Turku, Finland; 12 Institute of Molecular Medicine, University of Helsinki, Helsinki, Finland; Université de Montréal, Canada

## Abstract

**Objective:**

Serotonin transporter gene polymorphism (5-HTTLPR polymorphism) predicts the degree of structural and functional connectivity in the brain, and less consistently the degree of vulnerability for anxiety and depressive disorders. It is less known how 5-HTTLPR polymorphism influences on the coupling between brain and neuronal cardiovascular control. The present study demonstrates the impact of 5-HTTLPR polymorphism on the relations between heart rate (HR) corrected cardiac repolarization interval (QTc interval) and the brain 5-HTT binding.

**Material and Methods:**

Thirty healthy young adults (fifteen monozygotic twin pairs) (mean age 26±1.3 years, 16 females) were imagined with single-photon emission computed tomography (SPECT) using iodine-123 labeled 2β-carbomethoxy-3β-(4-iodophenyl) nortropane (nor-β-CIT). Continuous ECG recording was obtained from each participant at supine rest. Signal averaged QTc interval on continuous ECG was calculated and compared with the brain imaging results.

**Results:**

In the two groups [*l* homozygotes (n = 16, 10 females), *s* carriers (n = 14, 8 female)] HR and the length of QTc interval were not influenced by 5-HTTLPR polymorphism. There were no significant relations between HR and 5-HTT binding in the brain. There were significant associations between QTc interval and nor-β-CIT binding in the brain in *l* homozygotes, but not in *s* carriers (correlations for QTc interval and nor-β-CIT binding of striatum, thalamus and right temporal region were −0.8–−0.9, (p<0.0005), respectively).

**Conclusion:**

The finding of longer QTc interval with less 5-HTT binding availability in major serotonergic binding sites in *l* homozygotes, but not in s carriers, implicate to differentiated control of QTc interval by 5-HTTLPR polymorphism.

## Introduction

Serotonin transporter (5-HTT) is coded by a single gene (*SLC6A4*), which is located in human chromosome 17q11.2 [1]. After the discovery of polymorphism of the promoter region of the 5-HTT gene (5-HTTLPR polymorphism), with longer allele (with 16 repeats) having higher basal and induced transcription rates than shorter (with 14 repeats), the evidence is still inconclusive about the direct impact of 5-HTTLPR polymorphism on the 5-HTT binding in the human brain. Both significant associations and findings with no association have been reported [2] [3] [4] [5] [6] [7] [8].

Instead of direct gene effect on the brain 5-HTT binding, certain patterns of coupling of central nervous functions and structures are more clearly determined by 5-HTTLPR polymorphism. For example, there may be higher structural covariance between amygdala and anterior cingulate in individuals with both long alleles (*l* homozygotes) than in *s* allele carriers, and also there may be higher baseline amygdala activity with exaggerated responses to stressful images in *s* carriers than in *l* homozygotes [9] [10]. It has been hypothesized that 5-HTTLPR polymorphism causes these structural and functional variations by influencing on the timing and duration of 5-HTT gene expression [8].

In the present study young healthy adults were examined to assess the impact of 5-HTTLPR polymorphism on the functional integration in terms of relations between the brain 5-HTT binding and cardiovascular function, i.e. heart rate (HR), and heart rate corrected QT interval (QTc interval). We were particularly interested on the relations between QTc interval and brain nor-β-CIT binding, as we noticed the association between QTc interval and striatum nor-β-CIT binding in partially the same, but larger sample, but this finding remained without satisfactorial explanation [11]. The present study reanalyzes this interrelation with genotype data, which with functional connectivity context brings essentially new light to understand the relation. QTc interval is also particularly interesting compared with other measurements from ECG, because QTc interval is partially controlled by autonomic nervous system [12] [13].As *s* allele in 5-HTTLPR polymorphism has been related to less intra brain functional connectivity, we hypothesised similarly divergent pattern of integration between QTc interval and the brain 5-HTT binding by 5-HTTLPR polymorphism.

## Study Participants and Methods

### Study participants

The sample of thirty healthy Caucasian young adults from fifteen MZ pairs (mean age 26.2±1.3, range 21–28 years; 16 women), discordant or concordant for body mass (mean BMI 26±4, range 19–32 kg/m^2^), were recruited from the FinnTwin16 study born from 1975 to 1979. They were previously studied to explore metabolic and neurobiological correlates of obesity [14]. None of the study participants had a history of psychiatric disorder, substance abuse or eating disorder based on a structured psychiatric interview. Somatic health was checked by clinical examination (K.P), and by laboratory tests. Six women in three pairs used oral contraceptives, two persons were smokers, and seven reported weekly use of alcohol [14]. The study was approved by the ethical board of the Research Ethics Committee of Hospital district of Northern Savo and written informed consent was obtained from all participants.

### Genotyping

DNA was extracted from peripherally drawn blood samples from each study participants using standard methods. The SLC6A4 promoter region containing the long (16)/short (14) (l/s) polymorphism was PCR-amplified using the following primers: forward 5′-CGC TCC TGC ATC CCC CAT TA-3′ and reverse 5′-GGG ATG CGG GGG AAT ACT GGT-3′, which produced 297/253 bp (l/s) product. The genotype was analyzed in 3% MetaPhor (R) agarose (FMC BioProducts, Rockland, Maine) gel electrophoresis.

### Imaging procedure and data analysis

Single-photon emission tomography (SPECT), using iodine-123 labelled 2β-carbomethoxy-3β -(4-iodophenyl) nortropane (nor-β-CIT), was used for visualization and quantification of 5-HTT binding in the brain. Nor-β-CIT binds to monoamine transporters, both to 5-HTT and dopamine transporter (DAT) proteins, with ten-fold and 30% higher affinity to 5-HTT than DAT sites *in vitro* and *in vivo*, respectively. [15] [16]. As monoamine transporter density varies regionally in the brain, nor-β-CIT binding reflects predominantly DAT binding in striatum, and 5-HTT binding in midbrain, thalamus and cortical regions [17] [18] [19] [16]. Reported repeatability for high 5-HTT regions was good with 4% test-to-retest mean variation [20].

Each twin pair was studied on the same day before noon. Mean activity of injected nor-β-CIT CIT (supplied by MAP Medical Technologies Oy, Tikkakoski, Finland) was 195±5 MBq. Serial SPECT scans (with position lasers head position control) were performed at 5 min, 6 and 24 hours after the tracer injection using dedicated Siemens MultiSPECT 3 gamma camera equipped with fan-beam collimators (Siemens Medical Systems; Hoffman Estates, Ill., USA). The SPECT scans were decay-corrected and reconstructed with Butterworth-filtered back projection in a 128×128 matrix with a pixel size of 3×3 mm, and were attenuation-corrected with a Chang's algorithm. The imaging resolution was 8–9 mm. The SPECT slices were consecutively summarised to the slice thickness of 6 mm and re-aligned using a semi-automatic brain quantification program of Siemens and the Talairach coordinates. The slices were rotated and re-aligned so that transaxial (x-direction), sagittal (y-direction) and coronal (z-direction) ones were at right angles to each other. Region of interest placement was based on a semi-automatic brain quantification program of Siemens. The lower threshold of 60% of the maximum count was used to reduce the volume averaging and partial volume errors. Equilibrium analysis method was applied to estimate specific binding ratio (target-cerebellum)/cerebellum [21]. Peak equilibrium time was assumed to be 24 hours in the striatum and 6 hours in the midbrain and thalamus, temporal and midfrontal regions [22]. Analyses were done by experienced analyser who was blinded to other information about the study participants.

### ECG recording

After the brain imaging, continuous ECG recordings took place in a quiet dimly lit room during five minutes supine rest. AD -converted recordings were transferred into PC computer (digitalization with time resolution 200 Hz/channel) with WinCPRS program (Absolut Aliens, Turku, Finland). The Q wave was determined correspondingly as local minimum just before the R peak. The T-wave apex was determined as a local maximum between the S-peak and the P-wave. End of the T wave was determined as the crossing point of the isoelectric line and the tangent of the T-wave positioned at the half amplitude of the T-wave on the right side of the apex. The QT interval was determined as a time elapsed from the onset of Q wave to the end of the T wave, and steady state of 30–60 seconds of ECG recording was used for each sample of signal averaged QTc interval. For heart rate correction of QT interval Bazzet's, Friedricia's and Karjalainen approach were tested, and finally Karjalainen approach chosen [11]. (With Karjalanen approach QTc interval was independent on HR, which was not the case with Bazett's of Friedricia's corrections [23] [24].)

## Statistical Analysis

Results are presented as means ± SD. Mann-Whitney two-tailed tests were used for group comparisons of the data, when indicated. Pearson and Spearman correlations were used, when indicated. Using Bonferroni correction P<0.008 was taken to be statistical significant with a two tailed test. To identify those variables which are likely associated to QTc interval variation we employed linear regression analysis and multiple regression model, cluster-corrected to take into account the sampling of twins within twin pairs and thus provide correct standard errors and p-values [25]. SPSS 19.0 (SPSS Inc, Chicago, U.S.A.) and Stata (StataCorp, Texas, U.S.A.) programs were used for statistical analysis of the data.

## Results

Automatic detection of the QTc interval failed for three subjects (two *l* homozygotes and one *s* allele carrier). Basic characteristics of the study participants and nor-β-CIT binding values are presented in Tables 1 and 2. Means of body mass index, HR and QTc interval (n = 27) were not significantly different between *l* homozygotes and *s* carriers (Table 1). Mean thalamus 5-HTT binding was slightly lower in *l* homozygotes than in *s* carriers (Table 2).

**Table 1:** Basic characteristics in the two groups by 5-HTTLPR polymorphism.

	*l* homozygotes (n = 16)		*s* carriers (n = 14)	
	mean	SD	mean	SD
**Age (years)**	26	1.5	26	1.1
**BMI (kg/m2)**	24.9	3.5	26.6	3.6
**HR (bpm)**	67	11	62	17
**QTc (ms)**	354	15	345	17

QTc interval was available in thirteen *l* homozygotes and twelve s carriers. BMI = body mass index. None of the differences between the two genotype groups were statistically significant (p>0.05).

**Table 2:** Brain nor-β-CIT binding in the two groups by 5-HTTLPR polymorphism.

		Striatum	Thalamus	Midbrain	Tem (R)	Tem (L)	Midfront
***l homozygotes (16)***	Mean	2.65	1.09*	1.29	0.27	0.26	0.34
	SD	0.33	0.19	0.12	0.06	0.05	0.1
***s carriers (14)***	Mean	2.75	1.25*	1.36	0.28	0.27	0.31
	SD	0.28	0.17	0.18	0.09	0.11	0.15

* = Thalamus 5-HTT binding was slightly lower in *l* homozygotes compared to *s* carriers (p = 0.006). Tem (L/R) = left/right temporal region.

QTc interval was related to regional 5-HTT binding in *l* homozygotes, as presented in Table 3. There were no significant relations between 5-HTT binding and QTc interval in *s* carriers (Table 3). In *l* homozygotes gender, body mass index and QTc interval accounted for 48% and 61% of the variability in thalamic and the right temporal 5-HTT binding; for QTc interval; p = 0.001 and 0.0005, respectively (n = 14).

**Table 3:** Correlations between heart rate correlated QTc interval and brain nor-β-CIT binding in *l* homozygotes and *s* carriers.

		Striatum	Thalamus	Midbrain	Tem (R)	Tem (L)	Midfront
***l homozygotes***	r	−0.84**	−0.82**	−0.13	−0.87**	−0.70	−0.44
	P -value	<0.0005	<0.0005	0.67	<0.0005	0.01	0.11
***s*** ** carriers**	r	−0.56	−0.24	−0.13	0.16	−0.25	0.04
	P -value	0.04	0.44	0.66	0.60	0.40	0.90

** Correlation is significant at the level of 0.0005 (**) (two-tailed). QTc interval was available in fourteen *l* homozygotes and in thirteen *s* carriers, respectively.

## Discussion

The main finding of the present study suggests impact of 5-HTTLPR polymorphism on the relation between QTc interval and the brain 5-HTT binding; while QTc interval was significantly related to thalamus and temporal 5-HTT binding in *l* homozygotes, no association was found in *s* carriers.

There is some evidence for associations between cardiac repolarization and the brain function, linking QTc interval to brain and efferent autonomic physiology. In a large series of patients with prior brain infarction, the location of right or left insula for brain infarction was associated with abnormal cardiac repolarization [26]. Stellatum blockade in healthy individuals was associated with the length of QTc interval [13].

To our knowledge, the association between the brain 5-HTT binding and QTc interval is a novel finding. With a limitation of small sample size this relation is based on high repeatability of both QTc interval and 5-HTT binding in the thalamus [27] [20]. The association is most likely related to autonomic control of cardiovascular system, rather than other factors which regulate QTc interval. Parasympathetic blockade with atropine induced attenuation of QT shortening in HIS bundle paced dogs [28]. In humans electric stimulation of vagus nerve via auricular nerve induced similarly QT and HR shortening [12]. One study showed the effect of QTc prolongation along with significant HR increase with systemic sympathetic stimulation [29]. More detailed study showed prolongation of QTc interval after right stellatum blocade, and the opposite effect, after the left side blockade [13].

The relation between the brain 5-HTT binding and QTc interval was dictated by 5-HTT genotype in the present study. The influence of *s* allele was dominantly inhibited gene transcription *in vitro* [3]. Imaging studies have not been able to show direct gene effect from 5-HTTLPR to the brain 5-HTT-binding [8]. Still, there is robust evidence for the significance of 5-HTTLPR on the brain structure and function: increased amygdala activity, increased amygdala stress responsiveness, reduced gray matter volume in perigenual anterior cingulate cortex, diminished corticolimbic structural co-variation, and increased amygdala response for fearful stimuli has been reported in *s* carriers in comparison with *l* homozygotes [30]. We hypothesised that there might be heart-brain connection in this genotype dependent coupling, and our findings may widen the picture of 5-HTTLPR dependent functional integrity to encompass cardiac-brain axis. The influence of *s* allele was absence of cardiac-brain coupling.

## Limitations

This study is a preliminary evaluation with a small sample size (fourteen male and sixteen female participations) and generalizations from our findings should be taken with care.

The study participants were originally selected to evaluate metabolic neurobiological correlates of obesity, which is a limitation in principle, but adjustment for weight did not change the results.

Using bi-allelic gene characterization in this study may have influenced on the findings, but not likely towards the alternative hypothesis. The reported frequency of LGLA or LGLG genotype, obtained by tri-allelic gene characterization, is 10% in Finnish population, and it cannot be excluded that there was phenotypes with this genotype in the study participants [31].

We did not have superior accurate [11] C-DASB for 5-HTT imaging. Still, nor-β-CIT can be regarded as regionally specific tracer for 5-HTT imaging, and is has high reproducibility in high density regions of 5-HTTs, in the thalamus and in the midbrain [20].

## Conclusions

Our results suggest that in healthy young adults, who were dichotomized by 5-HTTLPR, there was significant relation between QTc interval and the brain 5-HTT binding only in *l* homozygotes. This may reflect divergent constitutional functional integration of *l* homozyogtes and *s* allele carriers by 5-HTTLPR.

## References

[1] Ramamoorthy S, Bauman AL, Moore KR, Han H, Yang-Feng T, et al (1993) Antidepressant- and cocaine-sensitive human serotonin transporter: Molecular cloning, expression, and chromosomal localization. Proceedings of the National Academy of Sciences of the United States of America 90: 2542–2546. 7681602 10.1073/pnas.90.6.2542 PMC46124

[2] Heils A, Teufel A, Petri S, Stober G, Riederer P, et al (1996) Allelic variation of human serotonin transporter gene expression. Journal of Neurochemistry 66: 2621–2624. 8632190 10.1046/j.1471-4159.1996.66062621.x

[3] Greenberg BD, Tolliver TJ, Huang SJ, Li Q, Bengel D, et al (1999) Genetic variation in the serotonin transporter promoter region affects serotonin uptake in human blood platelets. American Journal of Medical Genetics 88: 83–87. 10050973

[4] Heinz A, Jones DW, Mazzanti C, Goldman D, Ragan P, et al (2000) A relationship between serotonin transporter genotype and in vivo protein expression and alcohol neurotoxicity. Biol Psychiatry 47: 643–9. 10745057 10.1016/s0006-3223(99)00171-7

[5] van Dyck CH, Malison RT, Staley JK, Jacobsen LK, Seibyl JP, et al (2004) Central serotonin transporter availability measured with [123I]beta-CIT SPECT in relation to serotonin transporter genotype. Am J Psychiatry 161: 525–31. 14992979 10.1176/appi.ajp.161.3.525

[6] Parsey RV, Hastings RS, Oquendo MA, Huang YY, Simpson N, et al (2006) Lower serotonin transporter binding potential in the human brain during major depressive episodes. The American Journal of Psychiatry 163: 52–58. 16390889 10.1176/appi.ajp.163.1.52

[7] Praschak-Rieder N, Kennedy J, Wilson AA, Hussey D, Boovariwala A, et al (2007) Novel 5-HTTLPR allele associates with higher serotonin transporter binding in putamen: A [(11)C] DASB positron emission tomography study. Biological Psychiatry 62: 327–331. 17210141 10.1016/j.biopsych.2006.09.022

[8] Murthy NV, Selvaraj S, Cowen PJ, Bhagwagar Z, Riedel WJ, et al (2010) Serotonin transporter polymorphisms (SLC6A4 insertion/deletion and rs25531) do not affect the availability of 5-HTT to [11C] DASB binding in the living human brain. Neuro Image 52: 50–54. 20406689 10.1016/j.neuroimage.2010.04.032

[9] Meyer-Lindenberg A (2009) Neural connectivity as an intermediate phenotype: Brain networks under genetic control. Human Brain Mapping 30: 1938–1946. 19294651 10.1002/hbm.20639 PMC6871064

[10] Pezawas L, Meyer-Lindenberg A, Drabant EM, Verchinski BA, Munoz KE, et al (2005) 5-HTTLPR polymorphism impacts human cingulate-amygdala interactions: A genetic susceptibility mechanism for depression. Nature Neuroscience 8: 828–834. 15880108 10.1038/nn1463

[11] Kauppila E, Vanninen E, Kuusela T, Kaurijoki S, Karhunen L, et al (2009) Cardiac repolarization and striatal dopamine transporter function are interrelated. Nuclear Medicine Communications 10.1097/MNM.0b013e32832bdc96 19550362

[12] Zamotrinsky AV, Kondratiev B, de Jong JW (2001) Vagal neurostimulation in patients with coronary artery disease. Autonomic Neuroscience : Basic & Clinical 88: 109–116. 11474540 10.1016/S1566-0702(01)00227-2

[13] Egawa H, Okuda Y, Kitajima T, Minami J (2001) Assessment of QT interval and QT dispersion following stellate ganglion block using computerized measurements. Regional Anesthesia and Pain Medicine 26: 539–544. 11707793 10.1053/rapm.2001.25935

[14] Koskela AK, Kaurijoki S, Pietilainen KH, Karhunen L, Pesonen U, et al (2008) Serotonin transporter binding and acquired obesity – an imaging study of monozygotic twin pairs. Physiol Behav 93: 724–32. 18177905 10.1016/j.physbeh.2007.11.043

[15] Hiltunen J, Akerman KK, Kuikka JT, Bergstrom KA, Halldin C, et al (1998) Iodine-123 labeled nor-beta-CIT as a potential tracer for serotonin transporter imaging in the human brain with single-photon emission tomography. Eur J Nucl Med 25: 19–23. 9396870 10.1007/s002590050189

[16] de Win MM, Habraken JB, Reneman L, van den Brink W, den Heeten GJ, et al (2005) Validation of [(123)I]beta-CIT SPECT to assess serotonin transporters in vivo in humans: A double-blind, placebo-controlled, crossover study with the selective serotonin reuptake inhibitor citalopram. Neuropsychopharmacology 30: 996–1005. 15770240 10.1038/sj.npp.1300683

[17] Halldin C, Erixon-Lindroth N, Pauli S, Chou YH, Okubo Y, et al (2003) (11)C]PE2I: A highly selective radioligand for PET examination of the dopamine transporter in monkey and human brain. European Journal of Nuclear Medicine and Molecular Imaging 30: 1220–1230. 12811422 10.1007/s00259-003-1212-3

[18] Lundberg J, Odano I, Olsson H, Halldin C, Farde L (2005) Quantification of 11C-MADAM binding to the serotonin transporter in the human brain. Journal of Nuclear Medicine : Official Publication, Society of Nuclear Medicine 46: 1505–1515. 16157534

[19] Reneman L, Booij J, Lavalaye J, De Bruin K, De Wolff FA, et al (1999) Comparative in vivo study of iodine-123-labeled beta-CIT and nor-beta-CIT binding to serotonin transporters in rat brain. Synapse 34: 77–80. 10459174 10.1002/(SICI)1098-2396(199910)34:1<77::AID-SYN9>3.0.CO;2-Y

[20] Reneman L, Booij J, Habraken JB, De Bruin K, Hatzidimitriou G, et al (2002) Validity of [123I]beta-CIT SPECT in detecting MDMA-induced serotonergic neurotoxicity. Synapse 46: 199–205. 12325046 10.1002/syn.10130

[21] Acton PD, Kushner SA, Kung MP, Mozley PD, Plossl K, et al (1999) Simplified reference region model for the kinetic analysis of [99mTc]TRODAT-1 binding to dopamine transporters in nonhuman primates using single-photon emission tomography. European Journal of Nuclear Medicine 26: 518–526. 10382097 10.1007/s002590050420

[22] Hiltunen J, Åkerman K, Kuikka Jyrki T, Bergström KA, Halldin C, et al (1998) Iodine-123 labeled nor b-CIT as a potential tracer for serotonin transporter imagin in the human brain with single-photon emission tomography. European Journal of Nuclear Medicine 25: 19–22. 9396870 10.1007/s002590050189

[23] Bazett HC (1920) An analysis of the time-relations of electrocardiograms. Heart

[24] Karjalainen J, Viitasalo M, Manttari M, Manninen V (1994) Relation between QT intervals and heart rates from 40 to 120 beats/min in rest electrocardiograms of men and a simple method to adjust QT interval values. J Am Coll Cardiol 23: 1547–53. 8195512 10.1016/0735-1097(94)90654-8

[25] Williams RL (2000) A note on robust variance estimation for cluster-correlated data. Biometrics 56: 645–646. 10877330 10.1111/j.0006-341x.2000.00645.x

[26] Abboud H, Berroir S, Labreuche J, Orjuela K, Amarenco P, et al (2006) Insular involvement in brain infarction increases risk for cardiac arrhythmia and death. Annals of Neurology 59: 691–699. 16566012 10.1002/ana.20806

[27] Hojgaard MV, Holstein-Rathlou NH, Agner E, Kanters JK (2005) Reproducibility of heart rate variability, blood pressure variability and baroreceptor sensitivity during rest and head-up tilt. Blood Pressure Monitoring 10: 19–24. 15687870 10.1097/00126097-200502000-00005

[28] Nolan ER, Bailie MB, Olivier NB (2008) Effect of autonomic blockade on ventricular repolarization shortening: Response to behavioral stimulus in paced dogs. Autonomic Neuroscience : Basic & Clinical 140: 66–71. 18499531 10.1016/j.autneu.2008.04.004

[29] Magnano AR, Talathoti NB, Hallur R, Jurus DT, Dizon J, et al (2006) Effect of acute cocaine administration on the QTc interval of habitual users. The American Journal of Cardiology 97: 1244–1246. 16616034 10.1016/j.amjcard.2005.11.046

[30] Hariri AR, Drabant EM, Weinberger DR (2006) Imaging genetics: Perspectives from studies of genetically driven variation in serotonin function and corticolimbic affective processing. Biological Psychiatry 59: 888–897. 16442081 10.1016/j.biopsych.2005.11.005

[31] Hu XZ, Lipsky RH, Zhu G, Akhtar LA, Taubman J, et al (2006) Serotonin transporter promoter gain-of-function genotypes are linked to obsessive-compulsive disorder. American Journal of Human Genetics 78: 815–826. 16642437 10.1086/503850 PMC1474042

